# Design of a novel quantitative PCR (QPCR)-based protocol for genotyping mice carrying the neuroprotective Wallerian degeneration slow (*Wld*^*s*^) gene

**DOI:** 10.1186/1750-1326-2-21

**Published:** 2007-10-30

**Authors:** Thomas M Wishart, Stephen HF MacDonald, Philip E Chen, Michael J Shipston, Michael P Coleman, Thomas H Gillingwater, Richard R Ribchester

**Affiliations:** 1 Centre for Integrative Physiology, University of Edinburgh, Hugh Robson Building, George Square, Edinburgh, UK; 2 Centre for Neuroscience Research, University of Edinburgh, Hugh Robson Building, George Square, Edinburgh, UK; 3 Trinity Institute of Molecular Medicine, St. James's Hospital, Dublin 8, Ireland; 4 Laboratory of Molecular Signalling, Babraham Institute, Babraham, Cambridge, UK

## Abstract

**Background:**

Mice carrying the spontaneous genetic mutation known as Wallerian degeneration slow (*Wld*^*s*^) have a unique neuroprotective phenotype, where axonal and synaptic compartments of neurons are protected from degeneration following a wide variety of physical, toxic and inherited disease-inducing stimuli. This remarkable phenotype has been shown to delay onset and progression in several mouse models of neurodegenerative disease, suggesting that *Wld*^*s*^-mediated neuroprotection may assist in the identification of novel therapeutic targets. As a result, cross-breeding of *Wld*^*s *^mice with mouse models of neurodegenerative diseases is used increasingly to understand the roles of axon and synapse degeneration in disease. However, the phenotype shows strong gene-dose dependence so it is important to distinguish offspring that are homozygous or heterozygous for the mutation. Since the *Wld*^*s *^mutation comprises a triplication of a region already present in the mouse genome, the most stringent way to quantify the number of mutant *Wld*^*s *^alleles is using copy number. Current approaches to genotype *Wld*^*s *^mice are based on either Southern blots or pulsed field gel electrophoresis, neither of which are as rapid or efficient as quantitative PCR (QPCR).

**Results:**

We have developed a rapid, robust and efficient genotyping method for *Wld*^*s *^using QPCR. This approach differentiates, based on copy number, homozygous and heterozygous *Wld*^*s *^mice from wild-type mice and each other. We show that this approach can be used to genotype mice carrying the spontaneous *Wld*^*s *^mutation as well as animals expressing the *Wld*^*s *^transgene.

**Conclusion:**

We have developed a QPCR genotyping method that permits rapid and effective genotyping of *Wld*^*s *^copy number. This technique will be of particular benefit in studies where *Wld*^*s *^mice are cross-bred with other mouse models of neurodegenerative disease in order to understand the neuroprotective processes conferred by the *Wld*^*s *^mutation.

## Background

The Wallerian degeneration slow gene (*Wld*^*s*^) protects axons and synapses in both the CNS and PNS from injury-, neurotoxin- and inherited neurodegenerative conditions [1-5]. These properties have been shown to mitigate disease progression in several mouse models of neurodegenerative disease, including certain forms of motor neuron disease [[6,7]; but see [8]], gracile axonal dystrophy [9], Parkinson's disease [10], transient global cerebral ischemia [11], and myelin-related axonopathies [12]. Moreover, this remarkable neuroprotective phenotype has been transferred to other species, including rats and *Drosophila *[13,14]. The ability to deliver the *Wld*^*s *^gene to wild-type cells and confer neuroprotection across different species offers the possibility of developing *Wld*^*s*^-based therapeutics for treating human disease [5,13,15-17].

Evidence from studies of natural mutant mice and mice transgenic for *Wld*^*S *^suggests that its neuroprotective effect is strongly gene-dose dependent. For instance, reducing Wld^S ^protein expression by 50% removes almost all of the protective effect on motor nerve terminals while further reductions in the expression level additionally weakens the protection of distal axons (3, 18). As cross-breeding of *Wld*^*s *^mice with other mouse models of neurodegenerative disease becomes more commonplace, the ability to identify and distinguish wild-type from heterozygous and homozygous mice becomes more critical.

The *Wld*^*S *^mutation is a triplication of a region already present in the wild-type animal, encoding a fusion protein comprising the full length of nicotinamide mononucleotide adenylyltransferase 1 (Nmnat1; a NAD^+ ^synthesizing enzyme) coupled by a unique 18-amino acid sequence to the N-terminal 70 residues of the ubiquitination enzyme Ube4b [18,19]. Therefore, the means available to assess the number of mutant *Wld*^*S *^alleles present are by using either, the strength of the neuroprotective phenotype (for instance, based on protection of axons and synapses following a surgical nerve lesion) or detecting the copy number of the mutation. As well as an inefficient use of resources, the first approach is not suitable for genotyping on ethical and temporal grounds: an invasive procedure (nerve injury or biopsy) is required to test for strength of neuroprotection over a period of several days and provides only an indirect and semi-quantitative measure. The second approach is therefore the only one suitable for exact genotyping purposes.

Initially, attempts to PCR across the chimeric boundary were unreliable due to the very high GC content at that point in the sequence [20]. Some success was found through the use of Southern blots in identifying homozygous animals from heterozygous animals as it can be used to some extent to distinguish the genotype by band intensity [20]. However, Southern blotting proved to be an inefficient method for this type of genotyping as the results were often influenced by the need for equivalent loading concentrations, inconsistent DNA digestions, problems with blotting efficiency, and other factors that affected the quantifiable intensity of blots, their signal-noise ratio and the ability of the researcher to distinguish between samples on a film. A more robust method for genotyping *Wld*^*S *^mice was also reported by Mi and colleagues [20] based on Pulsed Field Gel Electrophoresis (PFGE). The PFGE method is based on fragment size rather than band intensity. Problems with this approach include the requirement for post-mortem material (not ideal for cross-breeding programs), time taken to obtain data and the requirement for the use of radio-labelled probes.

Here we report the development of a novel QPCR genotyping method that will allow rapid and effective genotyping of *Wld*^*s *^copy number.

## Results and discussion

### Quantitative PCR (QPCR) on genomic DNA for genotyping *Wld*^*s *^mice

C57BL/6J and C57BL/Wlds mice were obtained from Harlan UK and, where required were cross-bred to obtain F1 mice, heterozygous for Wlds. Ear punches were taken from mice between 1 and 4 months of age (N = 36 of confirmed genotype, N = 91 of unknown genotype). Tail tips were taken from transgenic rat lines Tg23 (N = 10) and Tg79 (N = 14). The investigator carrying out the QPCR screen was always blinded to the genotype of each tissue sample.

Genomic DNA was extracted using a modified proteinase K digestion/isopropanol precipitation protocol [21]. Tissue was incubated in tail lysis buffer (100 mM Tris-HCl, pH 8.5, 5 mM EDTA, 0.2% SDS, 200 mM NaCl, 100 μg/ml Proteinase K). Samples were incubated overnight at 55°C. Lysed tissue was vortexed for 1 minute and spun at 15800 *g *for 10 minutes to remove hair and bone. The supernatant was decanted into a new tube and isopropanol was added (equal volume to the tail lysis buffer used earlier). The solutions were inverted until a white precipitate was seen. The solution was centrifuged and the pelleted DNA was washed with 70% ethanol and air dried before it was resuspended in an appropriate volume of 1 × TE buffer. For example, a 5 mm length of tail tip was digested and the DNA was finally resuspended in 50 μl of TE buffer and then stored at -20°C as a stock solution.

**Figure 1:**
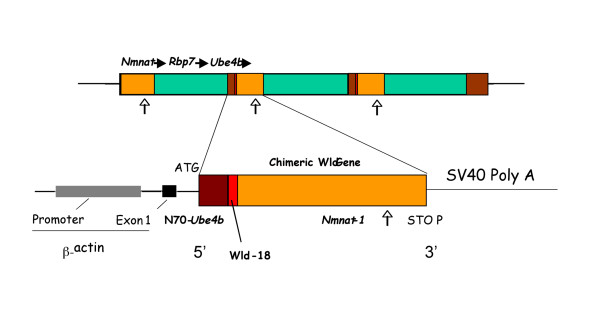
**The *Wld*^*s *^mutation and transgene**. The spontaneous mutation discovered in the *Wld*^*s *^mouse and the transgene used to make the various transgenic *Wld*^*s *^animals including Tg4836 mouse, Tg23 and Tg79 rat lines. The triplication results in the *Wld*^*s *^mouse having one full copy of *Nmnat1 *(shown in orange) and *Ube4b *(shown in brown) at either ends if the triplicated region. Within the triplicated region are 2 copies of an in frame fusion of the N70 amino acids of *Ube4b *linked to the whole coding region of *Nmnat1 *leading to the expression of 18 amino acids which are normally contained within the 5' untranslated region of *Nmnat1 *(termed Wld-18 shown in red) which has been used for antibody targeting. Primers against the *Wld*^*s *^sequence were designed to amplify bases 810–885 in the Genbank sequence AF260927 that correspond to a portion of the *Nmnat-1 *sequence in the chimeric *Wld*^*s *^product. Arrows indicate approximate site of amplification on the gene schematic. Figure modified from Gillingwater et al., 2006 [17].

The primers and probes (TaqMan Probes) were designed using Primer Express software as described in the ABI Primer Express User Bulletin (P/N 4317594). *Wld*^*s *^primers were based on Genbank entry AF260927 and recognise and amplify bases 810–885 of the *Wld*^*s *^chimeric gene (Figure 1). The *Wld*^*s *^probe was tagged with a FAM fluorophore and a TAMRA quencher. The product of the *Wld*^*s *^reaction is a 75 bp amplicon. β-Tubulin primers were based on Genbank entry M28739. The probe was tagged with a VIC fluorophore and a TAMRA quencher. The product of the β-Tubulin reaction is an 81 bp amplicon. The sequences (5'-3') used were as follows:

Wld^s ^forward GGCAGTGACGCTCAGAAATTC

Wld^s ^reverse GTTCACCAGGTGGATGTTGCT

Wld^s ^probe TCTACGAGTCCGATGTGCTGTGGAGACA

β-Tubulin forward GCCAGAGTGGTGCAGGAAATA

β-Tubulin reverse TCACCACGTCCAGGACAGAGT

β-Tubulin probe CTGGGCAAAGGGCCACTACACAGAGG

**Figure 2:**
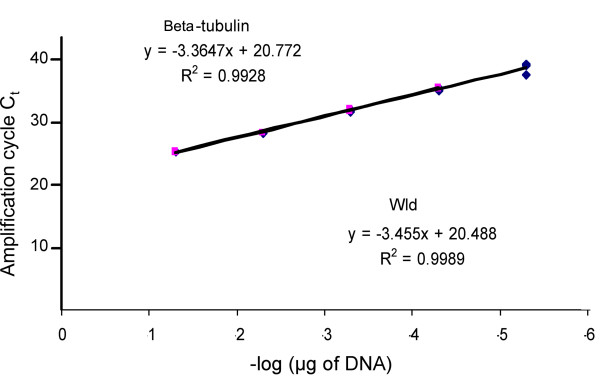
**Primers for *Wld*^*s *^and β-*tubulin *have very similar R^2 ^values**. Standard curves for the *Wld*^*s *^and β-*tubulin *gene in a serially diluted (1 in 10) template genomic DNA sample from wild-type (C57Bl/6J). Efficient amplification was obtained for both sets of primers, as demonstrated by the slopes of linear regression of the standard curves, and good correlation coefficients. C_t _is cycle threshold.

A serial dilution of genomic DNA from wild-type C57Bl/6J mice was used as a template for PCR to test the efficiency of amplification of the two primer pairs. The genomic DNA was diluted 1 in 10 each time and correlation coefficients were obtained for each primer pair (Figure 2). PCR efficiency was calculated by plotting the Threshold cycle (Ct) as a function of Log10 concentration of the template used (Figure 2; x-axis plotted as -Log μg DNA. See Applied Biosystems website for user bulletin #2 [22]). The slope of the trend line produced is a function of PCR efficiency, with a slope of -3.32 indicating close to 100% efficiency [22]. Standard curves suggested efficient amplification of both primer sets, as indicated by correlation coefficients and linear regression slopes. Primers against a region of *Wld*^*s *^and *β-tubulin *gave correlation coefficients of 0.9989 and 0.9928 respectively, with slopes of -3.455 for β-*tubulin *and -3.3647 for *Wld*^*s *^(Figure 2). The reactions were found to proceed similarly when primers were used both separately and together, indicating that the two separate primer pairs do not interact with each other. Once this had been demonstrated, genotyping reactions were reliably performed containing both sets of primers and probes in the same well.

The optimal primer concentrations used were 900 nM and the optimal probe concentration was found to be 250 nM. The PCR mix (TaqMan universal PCR master mix, no amperase UNG from ABI part # 4364341) was prepared according to the manufacturer's instructions. 1 μl of DNA was used at a concentration of 1:200 dilution (in TE buffer) of the original stock sample for each reaction. All reactions were carried out in triplicate on an ABI Prism 7000 sequence detection system using Applied Biosystems' standard thermal cycling parameters.

**Figure 3:**
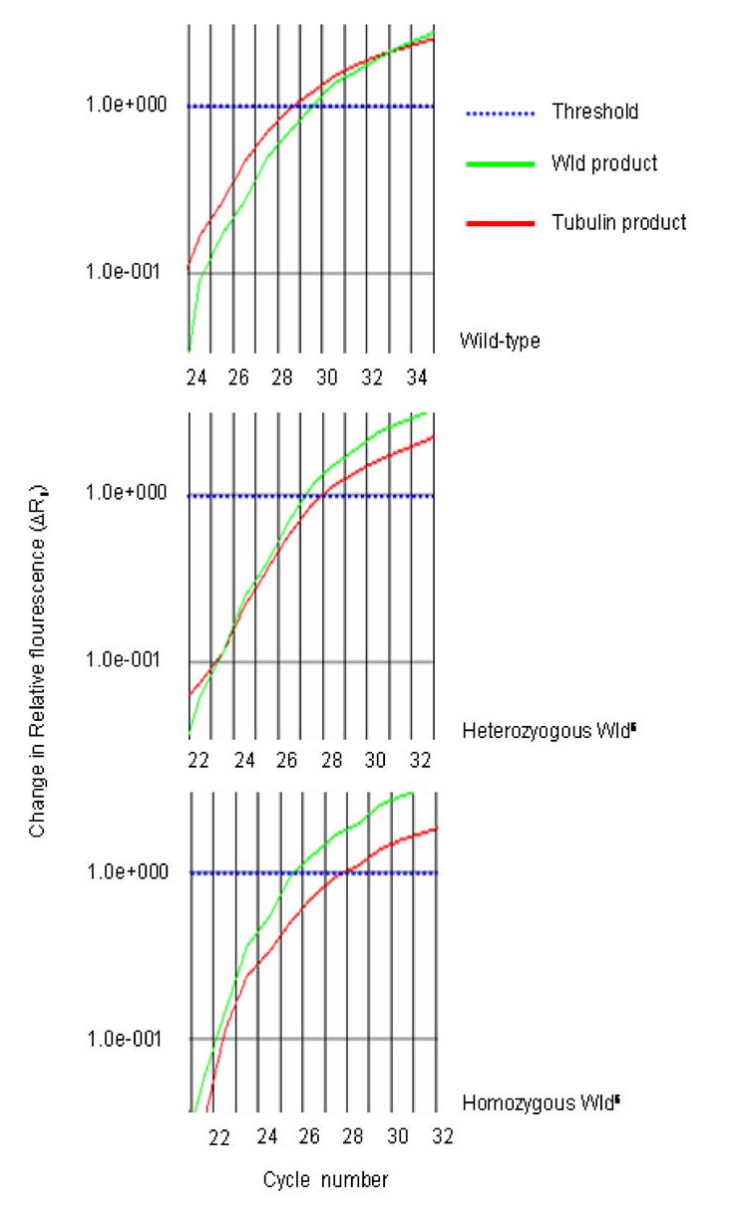
**Real time data shows differences between wild-type, heterozygous and homozygous animals**. Screen grabs from ABI software. The curves show the difference in cycle number (ΔC_t_) between tubulin (pseudo-coloured red) and Wld^s ^(pseudo-coloured green) for each genotype at the set threshold (1.0e+000, pseudo-coloured blue). These are representative curves only and should be viewed in conjunction with Figure 4.

**Figure 4:**
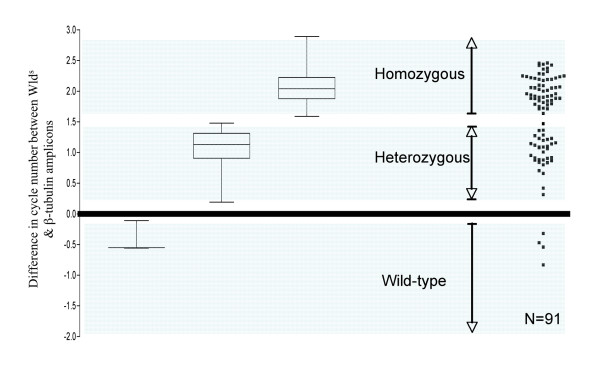
**QPCR on genomic DNA shows clear difference in ΔC_t _for the 3 genotypes**. A graphical representation of ΔC_t _between the tubulin and Wld amplicons, for animals of known genotype (N = 36, box and whisker) and animals of unknown genotype (N = 91, scatter). The areas shown in blue represent the 95% confidence limits for each particular genotype as determined from the box and whisker plots. There is a clear trend for each particular genotype.

The product of a PCR for the *Wld*^*s *^amplicon alone run on an ethidium bromide stained gel showed that bands from each genotype could not be distinguished from each other (data not shown). However, the 2^-ΔΔCT ^method (for more detail see Applied Biosystems website for user bulletin #2), using β-*tubulin *as an endogenous control and *Wld*^*s *^as a calibrator, allowed determination of copy number. Amplification plots for wild-type, heterozygous-*Wld*^*s *^and homozygous-*Wld*^*s *^showed clear differences for the ΔC_T _between each group (Figure 3 &4). The difference between β-*tubulin *and *Wld*^*s *^amplicon product at the set threshold can be used to determine *Wld*^*s *^genotype. A wild-type mouse gives a mean cycle difference of -0.40 (± 0.26 SD) with 95% confidence limits of -1.04 & +0.24. A mouse heterozygous for *Wld*^*s *^gives a mean cycle difference of 1.07 (± 0.32 SD) with 95% confidence limits of +0.97& +1.17. A mouse homozygous for *Wld*^*s *^gives mean cycle difference of 2.07 (± 0.30 SD) with 95% confidence limits of +01.995 & +2.158 (Figure 4).

Plotting the ΔC_t _against animals of known genotype in a box and whisker plot (N = 36) allows the illustration of 95% confidence limits for each genotype (Figure 4). When plotting a scatter of the results for all of the animals of unknown genotype (N = 91) there was a clear banding into the 3 genotypes as demonstrated by the box and whisker plot of animals of known genotype (Figure 4). Only one sample fell outwith the 95% confidence limits for all of the plotted genotypes shown (Figure 4). In such circumstances, the genotyping for this particular sample should be considered invalid. The *Wld*^*s *^status of a representative set of unknown genotype animals whose identity was determined by QPCR was successfully confirmed using breeding records, Southern blotting techniques and subsequent experiments where the neuroprotective phenotype was revealed by nerve lesion and the use of morphological techniques (data not shown).

### Determining copy number in transgenic animals

Transgenic animals (mice and rats) have been generated which express the *Wld*^*s *^chimeric gene [13,18]. These animals show a strong neuroprotective phenotype. As it has previously been shown that the *Wld*^*s *^neuroprotective phenotype is dose-dependent (see above), it is therefore of interest to determine the number of copies which have been integrated into the genome of these transgenic animals.

**Figure 5:**
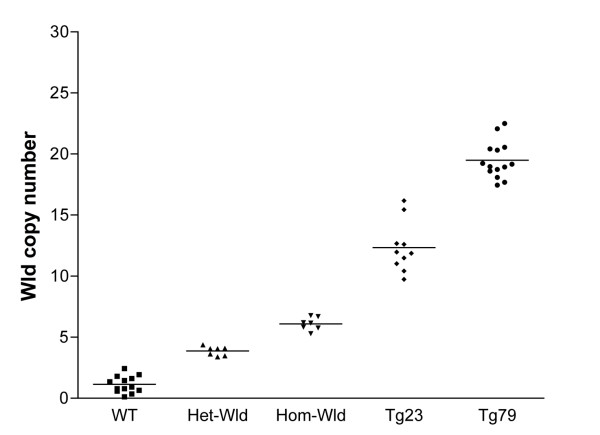
**QPCR can be used to determine copy number in transgenic animals**. QPCR carried out on samples of genomic DNA can be used to determine copy number of an insert in rat transgenic lines (Tg) 23 and 79. Although there is some fluctuation in the determined copy number, wild-type (WT) animals are expected to have 2 copies. Heterozygous *Wld*^*s *^(Het-Wld) are expected to have 4 copies and homozygous *Wld*^*s *^(Hom-Wld) should have 6 copies. Analysis of the QPCR data showed the following copy numbers: WT 1.15 ± 0.68 (mean ± SEM) copies (N = 13), Het-Wld 3.87 ± 0.36 copies (N = 7), Hom-Wld 6.10 ± 0.53 copies (N = 7), Tg23 12.34 ± 2.05 (N = 10) and Tg79 19.49 ± 1.52 (N = 14).

We examined 2 rat models of *Wld*^*s*^: the transgenic line 23 and transgenic line 79 [13]. Through the use of QPCR on genomic DNA (utilising the 2^-ΔΔCT ^method described above) it is possible to calculate the copy number of insertions into the rat genome. The results examining the number of inserts in two of the *Wld*^*s *^transgenic lines can be seen in Figure 5. There was additional variability in the amplification process at higher copy number with QPCR, as previously described when using this technology [22]. However, the data show that transgenic rat lines 23 and 79 contain 12.34 ± 2.05 (N = 10) and 19.49 ± 1.52 (N = 14) copies of the *Wld*^*s *^gene respectively, significantly more than homozygous *Wld*^*s *^mice, as would be expected in transgenic animals generated using non-targeted insertion. It is also important to note that the data do not give any indication of transgene orientation or functionality. Despite this, the increase *Wld*^*s *^copy number in the transgenic rats tallies with previously reported increases in phenotypic strength compared to homozygous *Wld*^*s *^mice [13].

It is especially important to measure *Wld*^*S *^copy number and expression level before making conclusions about its effects when attempting to transfer protective benefits to models of disease. For instance, two reports suggest little or no mitigation of disease onset or progression after crossbreeding *Wld*^*S *^mice with mouse models of amyotrophic lateral sclerosis (8,15); but it is presently unclear whether these reports fully accounted for the known, weak synaptic-protective effects of the *Wld*^*S *^gene in heterozygous and aged mice (3,18).

## Conclusion

The QPCR genotyping method we report here facilitates accurate, rapid and effective genotyping of *Wld*^*s *^copy number in both spontaneous mutant mice and transgenic animals expressing *Wld*^*s*^, representing an improved, cost effective and more efficient general purpose method than PFGE, the most accurate alternative method reported thus far. This methodology should be of interest to groups working on other mutations which are difficult to genotype as the DNA does not need to be accurately quantified before use. This is because the methodology works by examining ratios between PCR product of interest and controls rather than absolute levels. It is also applicable to smaller tissue samples such as ear punches. This technique will be of particular benefit for current and future studies where *Wld*^*s *^mice or their transgenic equivalents, are being crossed with other strains, and especially relevant for studies attempting to understand the relationship between gene-dosage of the *Wld*^*s *^mutation and the effectiveness of its neuroprotective phenotype.

## Competing interests

The author(s) declare that they have no competing interests.

## Authors' contributions

TMW designed and carried out the experiments, analysed data and drafted the manuscript. SHFM helped design primers and optimise reaction efficiency. PEC designed experiments. MPC generated and provided transgenic material. THG and RRR participated in study design and drafted the manuscript. All authors read and approved the final manuscript.
